# Single-Molecule Protein Fingerprinting with Photonic Hexagonal Boron Nitride Nanopores

**DOI:** 10.1021/accountsmr.3c00016

**Published:** 2023-03-14

**Authors:** Dong Hoon Shin, Xiliang Yang, Sabina Caneva

**Affiliations:** Department of Precision and Microsystems Engineering, Delft University of Technology, Mekelweg 2, 2628 CD, Delft, The Netherlands

## Introduction

Reading biomolecular signatures and understanding their role in health and disease is one of the greatest scientific challenges in modern biology. Decoding this information is not only foundational for biology but also a cornerstone for next generation molecular diagnostics. This calls for novel methods that can capture the presence and identity of low-abundance compounds at the individual molecule level. Since its inception in the 1980s, nanopore sequencing has become an essential part of the single-molecule sensing toolkit, proving that long, label-free reads of DNA can be achieved at low cost and high throughput.^1^ Despite their huge success in genome sequencing, reading the linear sequence of proteins is a considerably more complex task that requires differentiating 20 different amino acids (as opposed to the 4 DNA bases), as well as their modifications, during real-time translocation. Deconvoluting the time-dependent electrical current traces to determine entire amino acid sequences over long reads is as yet an unaccomplished milestone.

An alternative strategy is to identify proteins from partial sequence information obtained via highly sensitive optical readout schemes, which are well suited to parallel recordings of thousands of analytes simultaneously using wide-field imaging.^2^ Bioinformatics studies show that labeling a few subsets of amino acids is sufficient to enable identification of the majority of proteins in the human proteome,^3,4^ with more labeled subsets providing a less error-prone fingerprint. For instance, 90% of proteins can be correctly identified with reference to a proteomic database by the order in which labeled cysteine (C) and lysine (K) residues appear,^3^ while this number increases to ∼98% when C, K, and methionine (M) residues are labeled.^4^ This approach has been substantiated by single-molecule fluorescence based protein fingerprinting with biological nanopores.^5^ Attempting to sequence the cellular proteome in a time that is practically feasible, however, calls for techniques that offer higher throughput and robustness. Solid-state nanopores are attractive platforms, lending themselves to scalable production, and are thereby able to process thousands of single molecules simultaneously from a single device, while retaining high single-molecule resolution. We are developing monolithic photonic nanopores based on hexagonal boron nitride (hBN) crystals that directly integrate quantum emitters in the sensing region, paving the way for a single-molecule Förster resonance energy transfer (smFRET) detection scheme with a novel probe pair. On one hand, the platform can address fundamental questions on light–matter interactions in confined nanoscale volumes between solid-state and biological components, and on the other hand, hBN photonic nanopores have further research prospects in the detection of post-translational modifications (PTMs), which are important protein biomarkers of clinical utility. Due to their CMOS-compatible fabrication, they can be directly integrated with microfluidics, electronics, and photonics, and therefore represent a multifunctional and ultrasensitive analytical tool for future technologies in, for example, single-molecule liquid biopsy. Toward this aim, follow-up research directions include integration of sample preparation units to sort, enrich, and purify complex media (e.g., serum) in a fully on-chip workflow.

### Nanophotonic Biosensing with hBN

**Figure 1:**
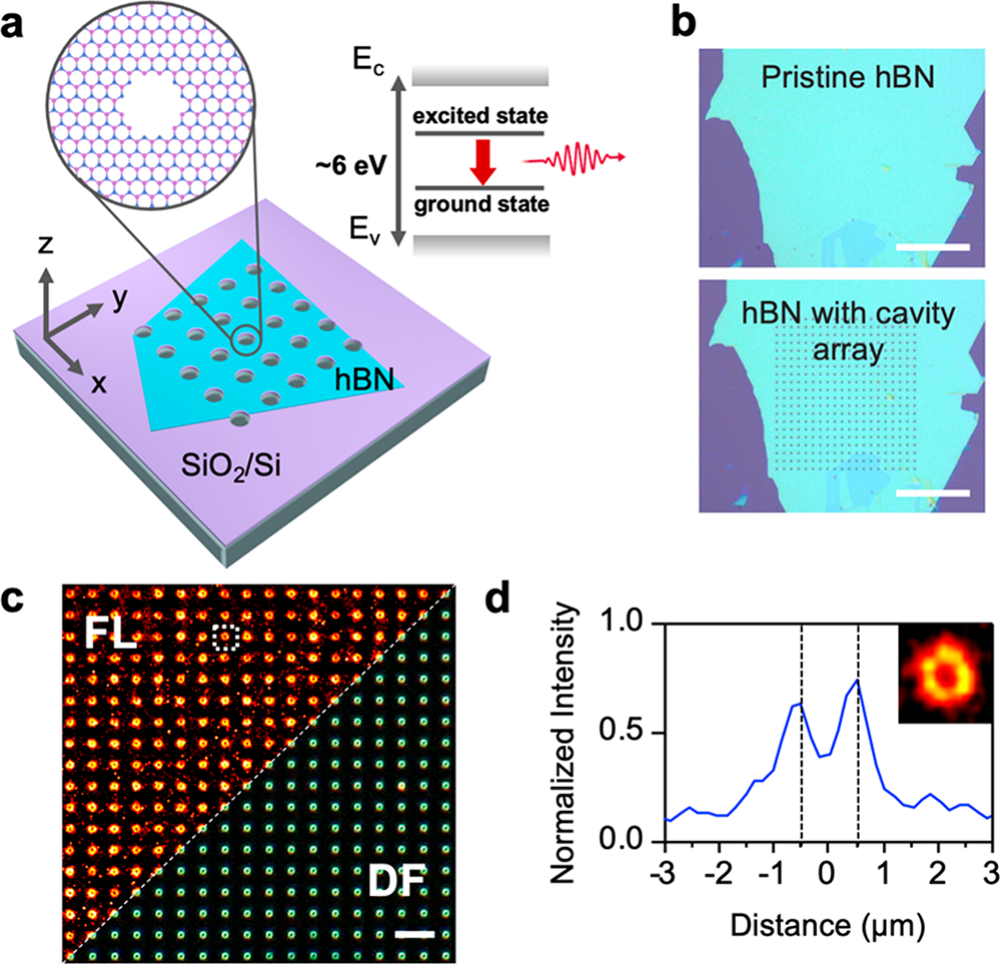
(a) Schematic of a cavity array fabricated in an hBN flake with crystal defects formed within the lattice (inset). Top-right inset: Simplified energy level diagram of an optically active defect in hBN with ground and excited states within the bandgap. (b) Optical images of an hBN flake before and after FIB milling. The scale bar is 50 μm. (c) Wide-field epifluorescence microscopy image (left, λ_ex_ = 532 nm) and dark-field optical microscopy image (right) of an hBN cavity array in Milli-Q water. The scale bar is 10 μm. (d) Line profile of the photoemission intensity from the hBN cavity in the dashed region in part c. Inset: zoomed-in image of the photoactive cavity.

Defects in materials can give rise to many intriguing physical properties. This holds particularly true for hBN, a wide bandgap 2D material, which hosts a broad range of deep-trap crystallographic defects. These defects can act as ultrabright (∼4000 kcts/s), highly photostable, room-temperature optical emitters,^6,7^ with experimentally determined quantum efficiencies of ∼87%, among the highest for solid-state emitters.^7^ Importantly, hBN optical defects can display narrow spectral line widths and maintain their outstanding photophysical properties in liquid and in harsh chemical environments,^8^ making them robust optical labels for applications in bioimaging/sensing under physiological conditions. The fluorescent lifetime (∼3 ns) also compares favorably with conventionally used organic dyes (∼0.3–1 ns).^7^ Endowed with this impressive range of photophysical properties, crystallographic hBN defects are being explored as near-perfect solid-state emitters for quantum sensing and super-resolution imaging of biomolecules. Such optically active emitters can be deterministically generated in hBN crystals using various techniques including focused ion beam (FIB) milling,^9^ electron irradiation,^10^ femtosecond (fs) laser ablation,^11^ plasma etching,^12^ and nanoindentation.^13^Figure 1a,b shows a proof-of-concept device in the form of a 20 × 20 cavity array produced by FIB milling in a mechanically exfoliated hBN flake and subsequently imaged with wide-field epifluorescence microscopy and dark-field optical microscopy (Figure 1c). The cavities exhibit strong photoemission at the rim under 532 nm illumination, indicating the presence of optically active defects (Figure 1d).

### Single-Molecule FRET Based Protein Fingerprinting

Energy transfer based sensing of biomolecules is a widely used scheme for fast and high-throughput bioimaging and biosensing. In this field, smFRET has become a popular tool for dynamic structural biology and a ubiquitous “spectroscopic ruler”, providing very accurate information about distances at the single-molecule level with high spatial (nanometer) and temporal (millisecond) resolution.^14^ In smFRET measurements, biomolecules of interest are optically labeled with a pair of probes (donors and acceptors). Non-radiative energy transfer takes place through dipole–dipole interactions between an excited donor and an acceptor, leading to changes in the fluorescence intensity and lifetime of the donor. Designing optimal probe pairs is thus a key enabling factor for the implementation of this technique, and smFRET measurements continue to evolve hand in hand with the development of donor and acceptor materials. In practice, the selection criteria for donors and acceptors are brightness, photostability, biocompatibility, room temperature operation, controllable chemistry, ease of production, and cost. Beyond organic dyes, a range of other materials are being explored as optical probes, including fluorescent proteins, inorganic nanoparticles, such as nanodiamonds and CdSe quantum dots, and more recently 2D materials.

Among those, 2D crystals of hBN are uniquely placed as a material fulfilling both the requirements of nanopore membranes and optical nanoprobes. We previously showed the implementation of hBN nanopores for DNA sensing, where geometrically defined pore shapes successfully enabled the distinction of DNA homopolymers.^15^ The combination of nanopore defect engineering and generation of on-chip nanopore arrays for biomolecule translocation ensures a high number of nanoscale confinement volumes, each with a built-in optical sensor.

**Figure 2:**
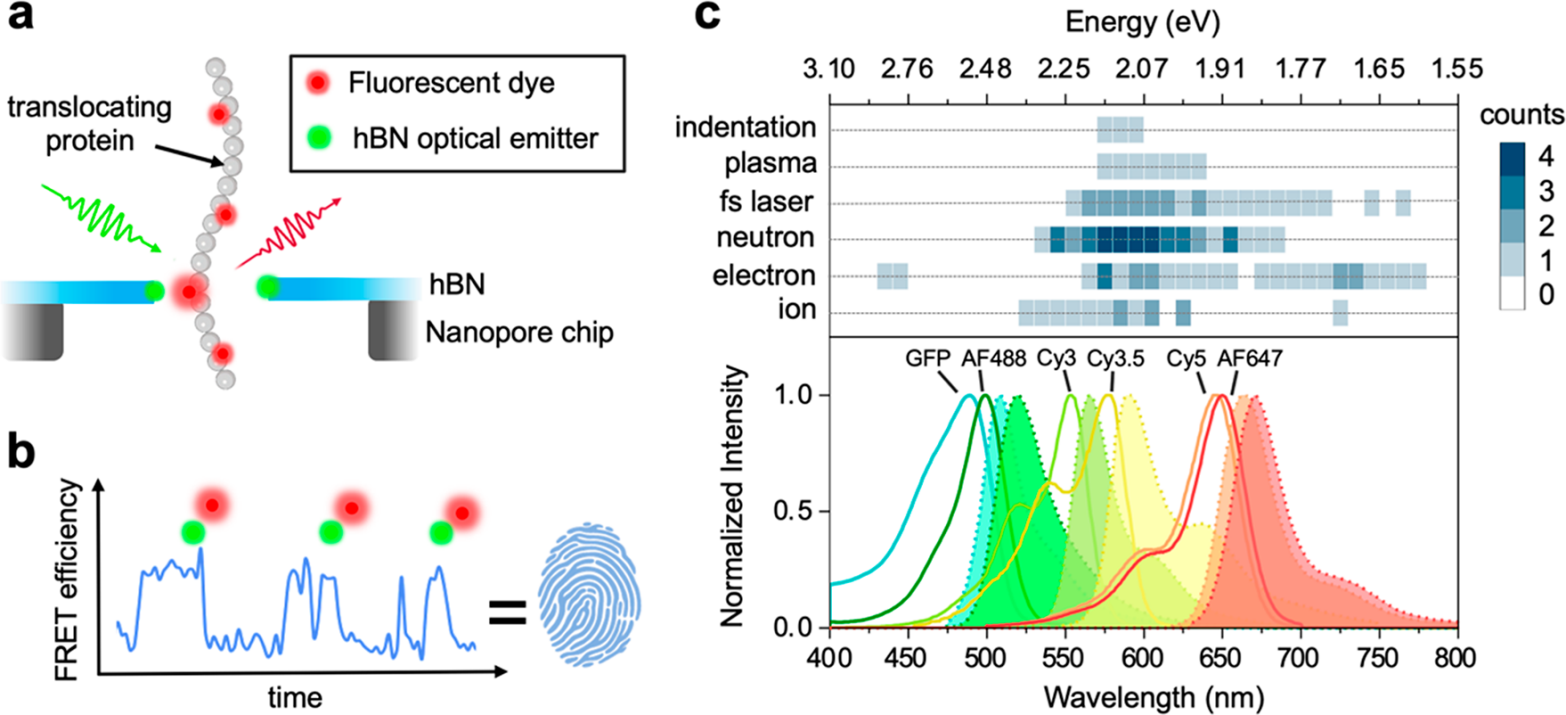
(a) Side view of single-molecule translocation through an hBN photonic nanopore with optical emitters in the rim. (b) Conceptual measurement of a single-molecule protein fingerprint generated by the sequence of high FRET states. smFRET occurs due to the interaction between an hBN optical emitter (green) and a labeled amino acid (red). (c) Upper panel: experimentally measured hBN ZPL energies generated using various techniques including nanoindentation, plasma treatment, fs laser ablation, neutron, electron, and ion irradiation methods. The emission ranges have been extracted from two comprehensive review papers,^7,17^ and their occurrence is shown by the intensity of the shaded region. Lower panel: the excitation and emission spectra for various types of fluorophores.

For single-protein analysis, we envisage that linearized proteins^16^ translocating through the hBN photonic nanopores pass in close proximity (<10 nm) to the hBN optical emitters located at the nanopore rim, giving rise to a high FRET signal. By monitoring the FRET efficiency as a function of time, the nanopore detects a sequence of high FRET states, indicating the passage of a specific labeled amino acid. The number of high FRET states and their separation in time (i.e., distance along the molecule) forms the basis of the biomolecule fingerprint (Figure 2a,b).

Unlocking the vast potential of hBN optical emitters for single-molecule nanopore fingerprinting is not without challenges. Exploiting these optical probes requires a better understanding of their spectral variability and, linked to this, the reproducible engineering and tuning of defect structures, since it is the defect crystal structure and composition (impurities, substitutional atoms, vacancies, and vacancy complexes)^7^ that dictate the photophysical properties and, by extension, the operation as a FRET probe. The top panel in Figure 2c shows the experimental zero phonon line (ZPL) energies of hBN optical emitters generated using various nanofabrication techniques.^7,17^ The lower panel shows the excitation and emission spectra of well-known fluorophores (GFP, AF488, Cy3, Cy3.5, Cy5, AF647), demonstrating their spectral compatibility as fluorescent labels for smFRET studies with several types of hBN emitters. This optical approach to single-molecule fingerprinting also relies on strategies to control the speed of (reversible) translocations in solid-state nanopores, which could include integration with optical, optoelectronic, magnetic, or acoustic tweezers.^14,18^

## Conclusion

The photonic nanopore platform presented here harnesses the outstanding properties of hBN optical emitters, which, by virtue of their high brightness in the visible and high quantum yield and photostability, represent promising nanoscale probes for fluorescence imaging of biomolecular features at the nanoscale. Specifically, solid-state nanopore transport measurements and single-molecule fluorescence time traces are combined to enable massively parallel and non-destructive protein fingerprinting. Given the capability to deterministically position and tune the optical properties of the emitters, hBN nanopores allow further opportunities for enhanced sensing, such as multicolor smFRET, thereby providing more complete and accurate protein fingerprints. Beyond protein identification, the platform could detect PTMs, which are of clinical relevance as biomarkers for molecular diagnostics. This nano-optofluidic platform can therefore signify an important step forward in enabling our understanding of the molecular details of proteins and their role in life.
